# Sublingual immunotherapy (SLIT) for house dust mites does not prevent new allergen sensitization and bronchial hyper-responsiveness in allergic rhinitis children

**DOI:** 10.1371/journal.pone.0182295

**Published:** 2017-08-14

**Authors:** Jae Hyun Lim, Jin Youp Kim, Doo Hee Han, Chul Hee Lee, Seung-No Hong, Jee Hye Wee, Sue K. Park, Chae-Seo Rhee

**Affiliations:** 1 Department of Otorhinolaryngology-Head and Neck Surgery, Seoul National University College of Medicine, Seoul National University Hospital, Seoul, Korea; 2 Department of Otorhinolaryngology-Head and Neck Surgery, Korea University College of Medicine, Korea University Ansan Hospital, Ansan, Korea; 3 Department of Otorhinolaryngology-Head and Neck Surgery, Bundang Jesaeng General Hospital, Deajin Medical Center, Seongnam, Korea; 4 Department of Preventive Medicine, Seoul National University College of Medicine, Seoul, Korea; 5 Department of Biomedical Science, Seoul National University Graduate School, Seoul, Korea; 6 Cancer Research Institute, Seoul National University, Seoul, Korea; 7 Department of Otorhinolaryngology-Head and Neck Surgery, Seoul National University College of Medicine, Seoul National University Bundang Hospital, Seongnam, Korea; 8 Institute of Allergy and Clinical Immunology, Seoul National University Medical Research Center, Seoul, Korea; 9 Sensory Organ Research Center, Seoul National University Medical Research Center, Seoul, Korea; Centre National de la Recherche Scientifique, FRANCE

## Abstract

**Introduction:**

The aim of this study is to identify the effects of sublingual immunotherapy (SLIT) on immunologic parameters and bronchial-hyper-responsiveness in children with allergic rhinitis to house-dust mite (HDM), through long-term follow-up cohort.

**Methods:**

Among the Allergic Rhinitis Cohort Study for Kids, pediatric patients who visited the hospital for rhinitis symptoms and proven allergy to HDM through skin prick test were studied. In this cohort, 37 patients received SLIT more than 3-years (SLIT group), and 184 patients received only pharmacologic therapy (non-SLIT group) were included in this study. The results of skin prick test, eosinophil percent and count, total immunoglobulin E (IgE), and bronchial provocation test at initial and 3-year followed-up were compared in the two groups.

**Results:**

After 3 year follow-up, only the serum eosinophil percent decreased more significantly in SLIT group than that in the non-SLIT group. New-sensitization rate other than HDM between SLIT and non-SLIT group did not show any significant differences. The distribution of sensitized allergen other than HDM showed increasing tendency after 3 years in both groups. Older age and a small number of sensitized allergen affected the improvement of bronchial hyper-responsiveness regardless of SLIT.

**Conclusion:**

HDM SLIT in allergic rhinitis children for 3 years in Korea does not affect prevention of new sensitization and poly-sensitization rate increment, and improvement of bronchial hyper-responsiveness.

## Introduction

Respiratory allergic diseases such as allergic rhinitis (AR) and asthma have been increased in recent years[1], both in developed and developing countries. Allergic rhinitis is characterized by nasal symptoms including nasal obstruction, rhinorrhea, sneezing and itching of the nose and by immunoglobulin E (IgE) mediated inflammation after exposure to specific allergens[2].

House-dust-mites (HDM) are most common allergens for AR in Korea[3], and allergen-specific immunotherapy is widely used for expectations that may modify the course of the disease. Subcutaneous or sublingual routes are mainly used methods for immunotherapy, there are no guidelines or standards on which to use. However, because of the concerns about adverse reactions related with subcutaneous immunotherapy such as local irritation or systemic reactions in rare cases, sublingual immunotherapy (SLIT) has been focused on a more secure method recently, especially patients with allergic rhinitis[4]. In many studies, including meta-analysis, have reported the clinical efficacy of SLIT to HDM for respiratory allergic disease, in terms of improving symptoms and reducing rescue medication use[5, 6].

However, there are only a few studies about immunologic effects of SLIT, and the results are still controversial even in double-blind-placebo-controlled studies. It has been reported that the HDM-specific IgE increased significantly in SLIT group compared to placebo group[7]. On the contrary, other study reported that the skin reaction to HDM, especially *Dermatophagoides pteronyssinus*, was significantly reduced in SLIT group compared to placebo group[8]. It was also reported there were lacks of changes in the immunologic parameters, in terms of HDM-specific IgE, IgG or IgG_4_[9]. This study is aimed to identify the effects of SLIT on immunologic parameters in children with allergic rhinitis based on data from Allergic Rhinitis Cohort Study for kids (ARCO-Kids).

## Materials and methods

### Study design

This study is based on data of pediatric patients who were enrolled ARCO-kids, a prospective cohort study that has been conducted since 2009. Children who visited in two tertiary hospitals (Seoul National University Hospital and Seoul National University Bundang Hospital) and sensitized to HDM in SPT performed at the time of enrollment were included in this study. All of the children (from 4 to 15 years old) had rhinitis symptoms (at least one of the nasal obstructions, rhinorrhea, sneezing, and itching sense of nose) and were assessed for allergic sensitivity using skin prick test (SPT) with inhalant allergens. Routine ear nose and throat (ENT) endoscopic examinations, serum total IgE, eosinophil percentage, SPT and/or methacholine bronchial provocation test were performed at initial and 3-year follow-up. At the same time, their parents were asked about children’s medical histories and completed the self-report questionnaires.

All the children had e received pharmacotherapy (oral medication and/or topical steroids) on demand with or without SLIT more than 3 years. According to their allergic treatment, children were grouped into SLIT group (treatment including SLIT) or non-SLIT group (treatment with pharmacotherapy alone)

A written informed consent was obtained from all patients or their parents. The Institutional Review Board of Seoul National University Bundang Hospital (IRB No. B-1311/228-101) and Seoul National University Hospital (IRB No. 1310-109-530) approved the study protocol.

### Immunologic tests

The serum level of eosinophil count (/uL), eosinophil percent (%), and total IgE (U/mL) were measured in each participant. Skin prick test, a total of 13 common standardized allergen extracts (SPT; Allergo-Pharma, Reinbek, Germany) was performed on the medial sides of both forearms. In our study, the allergens were categorized into 7 groups, depending on their characteristic: house-dust-mites (*Dermatophagoides pteronyssinus (Dp)*, *Dermatophagoides farinae (Df)*), molds (*Alternaria alternate*, *Aspergillus fumigates*), animal dander (cat, dog), cockroach (German cockroach), tree (mixture 1: alder, elm, hazel, poplar, and willow trees / mixture 2: beech, birch, oak, and plane trees), grass (mixture: velvet, orchard, rye, timothy, kentucky blue, meadow), weed (mugwort, ragweed). Histamine (1% of histamine phosphate) and 0.9% saline were used positive and negative controls, respectively. The number of sensitized allergens was calculated as the sum of positive results on these categorized allergens. The largest diameter of the wheal of each allergen was measured and interpreted as positive when 3 mm or more compare to negative control[10]. False positive or false negative cases were excluded. All patients were required to discontinue antihistamine or herbal medication at least 4 days before the test.

The methacholine provocation tests (MBPTs) were performed to the children equal or more than 6 years old. The bronchial hyperresponsiveness was defined when the provocative concentration below 16mg/mL caused a 20% decrease in FEV1 (PC_20_).

### Sublingual immunotherapy

For SLIT, standardized HDM extracts, Pangramin SLIT® (50% Dp/50% Df, ALK-Abello, Madrid, Spain), were used. For 30 days of escalation period, the patients increased the dose of administration as follows: 1 to 5 drops of 1.6 STU (specific treatment unit) /mL solution from days 1 to 10, 1 to 5 drops of 8 STU/mL solution from days 11 to 15, 1 to 5 drops of 40 STU/mL solution from days 16 to 20, 1 to 5 drops of 200 STU/mL solution from days 21 to 25, and 1 to 5 drops of 1000 STU/mL solution from days 26 to 30. After then, the allergen was maintained 5 drops of 1,000 STU/mL solution, 3 times per week. The patients were instructed to swallow after maintaining the drops for 2–3 minutes under their tongue.

### Total nasal symptom score (TNSS) and medication score

Patients’ nasal symptoms were investigated through self-report questionnaire which consisted of visual analogue scale of 0 to 10 cm (0: no complaints; 10: worst complaints) about rhinorrhea, sneezing, nasal obstruction, itchy nose, and eye discomfort. The total nasal symptom score was calculated by summing the score of each symptom.

The patients in SLIT group were requested to score the daily medications they took. Different points were given depending on the types of medication (1: oral or intranasal or ocular anti-histamines, 2: intranasal corticosteroid, 3: oral corticosteroids). The medication score was determined by summing the scores for one year.

### Statistical analysis

For analysis of laboratory data and continuous variables such as age and number of sensitized allergen, paired *t*-test or independent *t*-test were used within or between each group, respectively. All the continuous variables were presented as mean ± standard deviation (SD) unless otherwise specified. The categorical variables were analyzed using Chi-square or Fisher’s exact tests. To obtain the adjusted significance value, multivariate logistic regression analysis with the forward stepwise model was used. The changes in variables from initial to 3-year follow-up were analyzed between groups using repeated-measures analysis of variance (ANOVA). All significance tests conducted two-sided and *p*-value less than 0.05 were considered statistically significant. The SPSS software version 18.0 (SPSS Inc, Chicago, IL, USA) was used for statistical analyses.

## Results

### Study population

A total of 904 pediatric patients were enrolled in our cohort from 2009 to 2011. After excluding 186 patients with non-allergic rhinitis and 56 patients who sensitized to allergens other than HDM, 230 patients completed SPT at both initial and 3 years follow-up. Among them, 9 patients who received SLIT less than 3 years were additionally excluded, so finally, 221 pediatric patients were included in this study. Thirty-seven and 184 patients belong to SLIT group and non-SLIT group, respectively.

### General characteristics and immunologic factors

In the total study population, 154 patients were male and 67 patients were female. The mean age at initial evaluation was 7.7 ± 2.4 (range, 4–13) years old in the total population. SLIT group and non-SLIT group were 8.40 ± 2.05 and 7.59 ± 2.46 years old, respectively (P = 0.061). The mean duration from initial to follow-up tests were 37.2 ± 4.4 months. Initially, the wheal diameters of Dp and Df, and serum eosinophil percent in SLIT group were significantly larger than those in non-SLIT group (11.26 ± 4.13mm vs 8.23 ± 4.10mm, *P*<0.001; 9.32 ± 3.43mm vs 7.39 ± 3.29mm, *P* = 0.001; 7.41% ± 3.99% vs 5.75% ± 3.46%, *P* = 0.012; respectively). Three years later, the wheal diameters of Dp and Df in SLIT group were significantly larger than those in the non-SLIT group (10.70 ± 6.29mm vs 7.99 ± 3.55mm, *P* = 0.015; 9.91 ± 3.84mm vs 6.98 ± 2.52mm, *P*<0.001; respectively). These data are arranged in Table 1.

**Table 1:** Comparing the clinical features between SLIT and non-SLIT groups.

Variables	SLIT group (N = 37),	Non-SLIT group (N = 184)	*P*-value
**Initial**			
Age (year)	8.40 (range: 5–13)	7.59 (range: 4–13)	0.061
Number of sensitized allergen	1.51 ± 0.90	1.78 ± 1.05	0.149
Wheal diameter, Dp (mm)	11.26 ± 4.13	8.23 ± 4.10	<0.001*
Wheal diameter, Df (mm)	9.32 ± 3.43	7.39 ± 3.29	0.001*
Eosinophil percent	7.41 ± 3.99	5.75 ± 3.46	0.012*
Eosinophil count (/uL)	555.34 ± 374.02	440.55 ± 350.93	0.083
IgE (U/mL)	512.03 ± 427.64	385.55 ± 391.95	0.092
Number of bronchial hyper-responsiveness	10 (of 16 subjects)	31 (of 54 subjects)	0.716
**3-year follow-up**			
Number of sensitized allergen	2.03 ± 1.21	2.25 ± 1.51	0.398
Wheal diameter, Dp (mm)	10.70 ± 6.29	7.99 ± 3.55	0.015*
Wheal diameter, Df (mm)	9.91 ± 3.84	6.98 ± 2.52	<0.001*
Eosinophil percent	5.31 ± 3.05	5.92 ± 5.32	0.524
Eosinophil count (/uL)	367.26 ± 232.75	359.52 ± 221.42	0.862
IgE (U/mL)	444.35 ± 407.80	479.66 ± 636.84	0.762
Number of bronchial hyper-responsiveness	3 (of 16 subjects)	18 (of 54 subjects)	0.264

*: *P* <0.05 by independent t-test

Dp, *Dermatophagoides pteronyssinus*; Df, *Dermatophagoides farina*; IgE, immunoglobulin E

### Clinical efficacy of SLIT

TNSS in SLIT group was significantly decreased after 3-year follow-up (from 16.80 ± 8.99 to 12.72 ± 9.92, *P* = 0.026), while that in non-SLIT group was not significantly decreased (from 14.97 ± 8.85 to 13.12 ± 9.66, *P* = 0.078). However, the change of TNSS between SLIT and non-SLIT groups was not significantly different (-4.08 ± 8.61 vs -1.85 ± 12.21, *P* = 0.383; respectively). Medication score, recorded only in SLIT group, was significantly decreased after 3-year follow-up (from 229.14 ± 166.98 to 59.0 ± 117.5, *P*<0.001).

### Comparing the changes of immunologic factors

Comparing the changes of the number of sensitized allergen from initial to 3-year follow-up, both SLIT and non-SLIT groups were significantly increased (1.51±0.90 to 2.03±1.21, *P* = 0.016; 1.78±1.05 to 2.25±1.51, *P*<0.001; respectively), however, there was no significant difference between the two group (*P* = 0.853). The wheal diameters of Dp and Df were not significantly changed after 3 years in the both groups, and the changes were also not significantly different between SLIT and non-SLIT group. In terms of laboratory findings, serum eosinophil percent was significantly decreased only in SLIT group, and the change was significantly greater in SLIT group than that in the non-SLIT group (*P* = 0.015). There was no significant change in serum total IgE within each group and between groups (Table 2).

**Table 2:** Comparing the changes of SPT and serum laboratory data between SLIT and non-SLIT groups.

	SLIT group (N = 37)			Non-SLIT group (N = 184)			Between-group *P*-value
	Initial	3-year follow-up	Delta	Initial	3-year follow-up	Delta	
**Number of sensitized allergen**	1.51 ± 0.90	2.03 ± 1.21*	0.51 ± 1.24	1.78 ± 1.05	2.25 ± 1.51*	0.47 ± 1.40	0.853
**Wheal diameter, Dp (mm)**	11.26 ± 4.13	10.70 ± 6.29	-0.55 ± 5.80	8.23 ± 4.10	7.99 ± 3.55	-0.24 ± 3.75	0.676
**Wheal diameter, Df (mm)**	9.32 ± 3.43	9.91 ± 3.74	0.59 ± 3.85	7.39 ± 3.29	6.98 ± 2.52	-0.41 ± 3.73	0.139
**Eosinophil percent (%)**	7.41 ± 3.99	5.31 ± 3.05*	-2.44 ± 3.01	5.75 ± 3.46	5.92 ± 5.32	0.14 ± 5.72	0.015†
**Eosinophil count (/uL)**	555.34 ± 374.02	367.26 ± 232.75*	-219.01 ± 285.33	440.55 ± 350.93	359.52 ± 221.42*	-108.06 ± 403.94	0.153
**Total IgE (U/mL)**	512.03 ± 427.64	444.35 ± 407.80	-68.84 ± 234.50	385.55 ± 391.95	479.66 ± 636.84	71.78 ± 417.11	0.076

Delta was calculated by subtracting initial values from those of 3-year follow-up

*P-value < 0.05, within group (by paired *t*-test)

† *P*-value < 0.05, between groups (by repeated measures ANOVA)

### Reversion of HDM sensitization

Reversion (negative conversion after 3-year follow-up) of HDM-sensitized patients in 3-year follow-up SPT was revealed in 0 patient of SLIT group and in 6 patients (3.3%) of the non-SLIT group. In a non-SLIT group, reversion patients showed significantly lower initial immunologic values than those of non-reversion patients in terms of wheal diameters of Dp and Df, serum eosinophil percent and count, and serum total IgE. In multivariate analysis, only the serum eosinophil percent was significantly lower in reversion patients than that in a non-reversion patient (1.65% ± 0.51% vs. 5.89% ± 3.43%, *P* = 0.031; respectively). These data are arranged in Table 3.

**Table 3:** Analyzing the reversion cases of HDM sensitization in non-SLIT group.

Initial data	Reversion cases (N = 6)	Non-reversion cases (N = 178)	*P*-value, crude	*P*-value, adjusted
**Age**	7.33 ± 1.51	7.60 ± 2.48	0.794	0.980
**Number of sensitized allergen**	2.00 ± 2.00	1.78 ± 1.02	0.609	0.716
**Wheal diameter, Dp (mm)**	3.75 ± 2.25	8.38 ± 4.06	0.006*	0.156
**Wheal diameter, Df (mm)**	3.54 ± 3.20	7.52 ± 3.22	0.003*	0.130
**Eosinophil percent (%)**	1.65 ± 0.51	5.89 ± 3.43	<0.001*	0.031†
**Eosinophil count (/uL)**	130.40 ± 64.07	449.95 ± 351.83	0.045*	0.992
**Total IgE (U/mL)**	56.20 ± 38.50	395.53 ± 393.53	<0.001*	0.356

Reversion means the change of reaction from positive in the initial test to negative in the 3-year follow-up test.

* p < 0.05, by independent *t*-test

† p < 0.05, by logistic regression test

### Development of new sensitization in HDM mono-sensitization cases

Initially, there were 22 and 97 patients with mono-sensitization to HDM (Dp or Df) only in SLIT and non-SLIT group, respectively. In SLIT group, the initial age, wheal diameter of HDM (*Dp*, *Df*), and serum total IgE level were significantly higher than those of non-SLIT group. Among them, 12 (54.5%) and 38 (39.2%) patients became sensitized to new allergens after 3-year follow-up in SLIT and non-SLIT group, respectively, and the rates of new sensitization were not significantly different between the two groups. These data are arranged in Table 4.

**Table 4:** Analysis of the patients with mono-sensitization to HDM at initial: General characteristics, immunologic data, and new-sensitization rate after 3-year follow-up.

General characteristics (initial values)	SLIT group (n = 22)	Non-SLIT group (n = 97)	*P*-value
Age (years)	8.50±2.06	6.81±2.23	0.001*
Sex (M:F)	16:6	58:35	0.461
Wheal diameter, Dp (mm)	11.20±4.27	7.15±3.93	<0.001*
Wheal diameter, Df (mm)	9.09±3.66	6.67±2.99	0.001*
Eosinophil percent (%)	6.47±5.45	5.30±3.57	0.188
Eosinophil count (/uL)	491.24±393.67	382.02±272.03	0.133
Total IgE (U/mL)	481.64±306.27	294.44±350.32	0.037*
**New-sensitization after 3 years**	12 (54.5%)	38 (39.2%)	0.187 (0.348†)
Molds	4	7	
Animal dander	8	20	
Cockroach	2	6	
Tree	3	16	
Grass	0	12	
Weed	4	12	

* *P* < 0.05, by independent *t*-test

† Adjusted *P*-value for age, sex, wheal diameter of Dp and Df, and total IgE, by logistic regression analysis

The numbers are not mutually exclusive.

### Development of new sensitization in HDM poly-sensitization cases

Poly-sensitization was defined as sensitization to HDM (Dp or Df) plus other allergens. Initially, there were 15 and 87 patients with poly-sensitization in SLIT and non-SLIT group, respectively. In SLIT group, the initial eosinophil percent was significantly higher than those of non-SLIT group. Three years later, 4 (26.7%) and 39 (44.8%) patients became sensitized to new allergens in SLIT and non-SLIT group, respectively, and the rates of new sensitization were not significantly different between the two groups. (Table 5).

**Table 5:** Analysis of the patients with poly-sensitization including HDM at initial: General characteristics, immunologic data, and new-sensitization rate after 3-year follow-up.

General characteristics (initial values)	SLIT group (n = 15)	Non-SLIT group (n = 87)	*P*-value
Age (years)	8.27±2.09	8.48±2.41	0.761
Sex (M:F)	13:2	64:21	0.510
No. of sensitized allergen	2.27±1.03	2.62±0.89	0.164
Wheal diameter, Dp (mm)	11.33±4.06	9.44±3.96	0.111
Wheal diameter, Df (mm)	9.65±3.14	8.19±3.44	0.158
Eosinophil percent (%)	8.83±3.58	6.26±3.27	0.011*
Eosinophil count (/uL)	651.50±332.93	506.39±414.48	0.218
Total IgE (U/mL)	561.11±446.30	492.71±413.17	0.586
**New-sensitization after 3 years**	4 (26.7%)	39 (44.8%)	0.385 (0.229†)
Molds	0	5	
Animal dander	1	15	
Cockroach	1	7	
Tree	3	10	
Grass	2	13	
Weed	0	15	

* *P* < 0.05, by independent t-test

† Adjusted *P*-value for age, sex, and eosinophil percent, by logistic regression analysis.

The numbers are not mutually exclusive.

### Distribution of sensitized allergens

**Fig 1:**
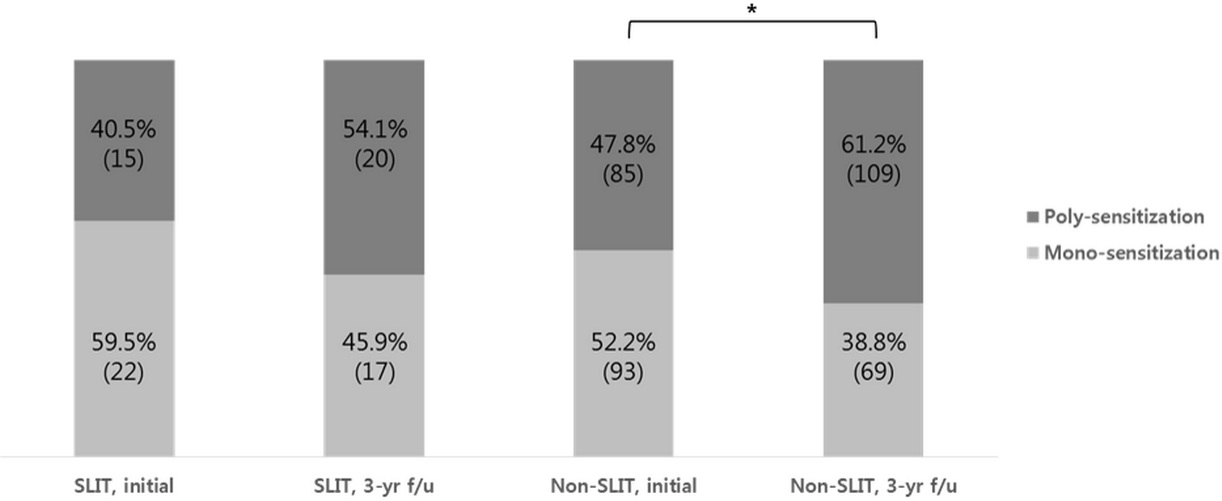
The percentage of mono- or poly-sensitization cases between SLIT and non-SLIT groups. The poly-sensitization rates were increased in both (SLIT and non-SLIT) groups, and the increment was significant in non-SLIT group.* *P* < 0.05, by Chi-square test.

In SLIT and non-SLIT group, the incidence of poly-sensitization was increased after 3 years, from 40.5% to 54.1% (*P* = 0.244) and from 47.8% to 61.2% (*P* = 0.011), respectively. There was no significant difference of poly-sensitization rate between SLIT and non-SLIT group at the initial (*P* = 0.424) and 3-year follow-up (*P* = 0.417) (Fig 1). The sensitizations to individual allergens other than HDM were also increased in two groups, mainly animal dander, tree, grass, and weed pollens, and their increments showed significances especially in a non-SLIT group. (Table 6)

**Table 6:** Distribution of individual allergen sensitization other than HDM.

Allergen	SLIT group (N = 37)			Non-SLIT group (N = 184)		
	Initial	3-year follow-up	*P*-value	Initial	3-year follow-up	*P*-value
**Molds**	6 (16.2%)	7 (18.9%)	0.760	31 (16.8%)	34 (18.5%)	0.682
**Animal dander**	6 (16.2%)	12 (32.4%)	0.104	42 (22.8%)	63 (34.2%)	0.021*
**Cockroach**	1 (2.7%)	4 (10.8%)	0.358	13 (7.1%)	17 (9.2%)	0.446
**Tree**	2 (5.4%)	8 (21.6%)	0.085	30 (16.3%)	52 (28.3%)	0.006*
**Grass**	1 (2.7%)	2 (5.4%)	1.000	9 (4.9%)	33 (17.9%)	< 0.001*
**Weed**	3 (8.1%)	5 (13.5%)	0.711	19 (10.3%)	37 (20.1%)	0.009*

* *P* < 0.05, by Pearson’s chi-square

### Incidence of bronchial hyper-responsiveness

**Fig 2:**
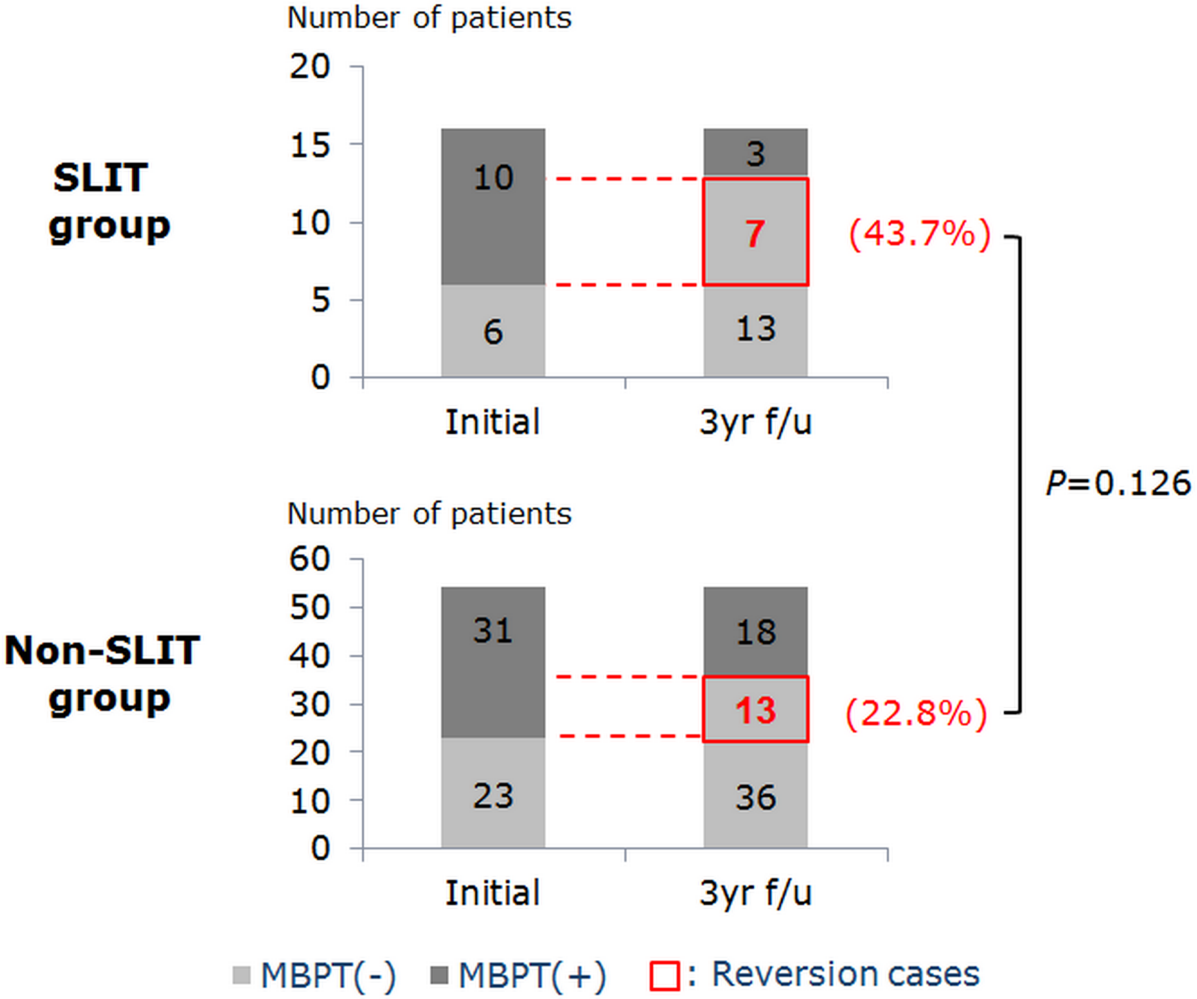
Changes in the incidence of bronchial hyper-responsiveness after 3-year follow-up. Reversion rate of bronchial hyper-responsiveness were not significantly decreased after 3-year of SLIT compared with that of non-SLIT group. MBPT: methacholine bronchial provocation test. Reversion means the change of reaction from positive in the initial test to negative in the 3-year follow-up test.

A total of 70 patients who complete MBPT at initial and 3-year follow-up, 16 patients were SLIT group and 54 patients were a non-SLIT group. In SLIT group, 3 patients were positive and 6 patients were a negative reaction at both initial and 3-year follow-up test. In a non-SLIT group, 18 patients were positive and 23 were a negative reaction at both initial and 3-year follow-up test. The reversion cases, who were initially positive but became negative reaction at 3-year follow-up MBPT, were 7 (43.7%) and 13 (22.8%) patients in SLIT and non-SLIT group, respectively, without statistical significance (OR = 2.453, 95% CI 0.763–7.890; *P =* 0.126) (Fig 2). When analyzed together with the other contributing factors that can affect bronchial hyper-responsiveness, older age and a smaller number of sensitized allergen have been shown to be related with the reversion of bronchial hyper-responsiveness regardless of SLIT (Table 7). There were no positive conversion cases of bronchial hyper-responsiveness.

**Table 7:** Analyzing the reversion cases of bronchial hyper-responsiveness.

Initial data	Reversion group (N = 20)	Non-reversion group (N = 21)	*P*-value, crude	*P*-value, adjusted
**Age**	9.45 ± 1.79	8.24 ± 1.51	0.024*	0.019†
**Number of sensitized allergen**	1.65 ± 0.88	2.52 ± 1.60	0.037*	0.040†
**Wheal diameter, Dp (mm)**	10.70 ± 4.05	10.68 ± 4.74	0.988	0.947
**Wheal diameter, Df (mm)**	9.05 ± 3.49	8.11 ± 3.87	0.418	0.467
**Eosinophil percent**	7.13 ± 3.54	6.69 ± 4.22	0.721	0.870
**Eosinophil count (/uL)**	508.85 ± 318.76	491.35 ± 347.72	0.869	0.935
**Total IgE (U/mL)**	342.22 ± 305.96	533.49 ± 445.53	0.128	0.253
**SLIT (n)**	7	13	0.126	0.928

Reversion means the change of reaction from positive in the initial test to negative in the 3-year follow-up test.

* *P* < 0.05, by independent t-test

† *P* < 0.05, by logistic regression test

## Discussion

The main mechanism of immunologic modification by allergen-specific immunotherapy, so-called allergen tolerance, is understood as peripheral T-cell tolerance characterized by the generation of CD4^+^CD25^+^ regulatory T (T_reg_) cells[11–13]. T_reg_ cells produce cytokines that have regulatory activities, Interleukin (IL)-10 and transforming growth factor-β (TGF-β), and they play an important role in suppressing the allergic inflammation by inhibiting type 2 helper T cells as well as mast cells, basophils, and eosinophils[12, 14]. According to this theory, many immunologic factors that reflect allergic reaction should be decreased after SLIT, but there have been many controversies in clinical results about them[7–9]. Our study has strengths in respect of prospectively designed cohort based study, and using immunologic markers that are important, easily accessible, and commonly used in clinical practice. In this study, retrospective analysis was performed without matching the participants’ age and sex, because we wanted to investigate retrospectively the changes of many immunologic parameters from prospective cohort. To overcome this limitation, confound factors including age and sex were adjusted when comparing the proportion of new-sensitization between SLIT and non-SLIT group.

In our results, among the skin reaction to HDM at SPT (wheal diameter), serum eosinophil percent, and serum total IgE, only eosinophil percent showed significantly decreased in SLIT group. There are some controversial investigations with different results about these parameters, and exact immune-mediated reaction by SLIT is still unclear. In the results on skin sensitivity or reaction, it has been reported that the wheal diameter was significantly decreased after treatment of SLIT, even in double blinded placebo controlled studies[8, 15–18]. Theoretically, the effect of inhibiting skin reaction is supported by the action of IL-10, and IgG_4_ and IgA antibodies induced by immunotherapy[19]. Since our SLIT dose did not differ from other previous studies[15, 18] and patients’ compliance was good, we are curious about the lack of inhibitory effect to skin reaction in the present study. Although it is difficult to give a clear and proper explanation, there are also some studied that reported the same results as ours[20, 21]. In terms of the serum IgE, some studies, in which specific IgE was measured over time during antigen specific immunotherapy, reported that the specific IgE initially increased and then returned to its pretreatment level after 4 weeks of treatment.[19, 22] The total serum IgE showed similar course[22], so it did not show significant difference after SLIT in long-term follow-up studies[15, 16, 18, 23]. Our study investigated total IgE only, since the test for specific IgE was not performed on all patients, and it showed no significant change after 3-year follow-up in both groups. On the other hand, there are some tendencies of improvements in eosinophil level or its activity, such as eosinophil count or eosinophil cationic protein [24], by SLIT. Our results also support these trends. Interestingly, the eosinophil percent at the initial test was significantly lower than that of reversion cases. It can suggest that the natural course for reversion from HDM could be related with low initial eosinophil percent, so the tendency of reduction in eosinophil percent by 3-year of SLIT might have a potential suppression effect on HDM sensitization. However, there are no reversion cases in SLIT group, maybe due to a small number of patients and significantly higher level of initial eosinophil percent than that in the non-SLIT group, so we could not analyze the reversion cases between SLIT and non-SLIT group. We tried to find out the cut-off value of eosinophil percent that can predict reversion, however, there are no definite values that can differentiate reversion from non-reversion cases (AUC 0.072 in ROC curve).

Generally, the clinical outcome is more emphasized than objective parameters for evaluating the efficacy of SLIT.[25, 26] In our study, the clinical efficacy was evaluated by both TNSS and medication score which were significantly decreased in SLIT group. In non-SLIT group, the TNSS showed a near-significant decrease (*P*-value = 0.078). The reason why the improvement of TNSS in the non-SLIT group was not inferior to that in the SLIT group could be explained by the fact that the patients in the non-SLIT group had taken medication regularly or on their demands. The exact medication scores were not surveyed in the non-SLIT group, since they were regularly controlled by medication, and we assumed that their medication scores could not be changed with time. However, it is a limitation that the comparison of medication score between the two groups cannot be done, so that the clinical improvement with SLIT was observed but could not be completely verified.

Several studies have revealed that allergen-specific immunotherapy has potential to prevent new-sensitization in allergic rhinitis and/or asthma patients who mono-sensitized to HDM. In a study of asthmatic children in France, the new sensitization occurred in all of the 22 control group (non-SLIT) but in 10 of the 22 SLIT group (*P*<0.001) after 3-year observation[27]. In another study of asthma patients with a larger sample and longer follow-up period (6-year) in Italy, 36 of the 54 (66.7%) control group showed new sensitization in compared with 17 of the 69 (24.6%) SLIT group (*P*<0.0002)[28]. The other study, conducted in Turkey, of allergic rhinitis and/or asthma patients also reported same result (33 of the 62 control group vs. 11 of the 86 SLIT group, *P* = 0.002)[29]. All of these studies were prospectively designed, all patients were sensitized to HDM alone, and allergen-specific immunotherapy was performed with subcutaneous injection. In contrast to these previous results, the present study cannot show the preventive effect of SLIT for new sensitization not only in mono-sensitization to HDM group but also in the poly-sensitization group. It may due to some different points previous and this present study. First, all the previous studies were conducted in Western countries while this study was done in Korea, therefore, the environmental and genetic predisposition[30] may influence the allergic reaction different from previous studies. Second, SLIT was used for immunotherapy instead of SCIT in this study. Although the mechanisms of immunologic tolerance between the two modalities are known to be similar[31], some differences have been reported also[18, 32]. Finally, there were some significant differences in general characteristics of SLIT compared with those of non-SLIT group; older age, larger wheal diameter of HDM, and higher total IgE level. In the previous studies, the patients’ age did not show significant differences. Age is a possible contributing factor that may influence allergen sensitization, indeed, there are some studies that the prevalence of poly-sensitivity was increased with age in children[33, 34]. The effect of wheal diameter of HDM to other allergen sensitivity has not been evaluated yet, however, it can be hypothesized that high HDM skin sensitivity and total IgE level represent more severe profiles of allergic reaction, accordingly, may affect the sensitivities to other allergens.

In children, generally, the prevalence of sensitization for individual allergens is known to increase so that the poly-sensitization rate is increased with age[3, 33]. Similar to these previous reports, the poly-sensitization rate and distribution of individual allergens in the non-SLIT group tended to increase after 3-year follow-up, especially in animal dander, tree, grass, and weed, they were significantly increased. It seems that SLIT did not prevent this trend sufficiently because the allergen sensitizations showed increasing tendency even though it was not statistically significant.

Several studies revealed that SLIT could reduce bronchial hyper-responsiveness with improved values of pulmonary function such as FVC, FEV1, and PEF.[2, 17, 23] While our study did not present the effect of SLIT regarding negative conversion of bronchial hyper-responsiveness after 3-year treatment, older age and small number of sensitized allergen at initial test was related to higher negative conversion rate. In the recent research performed in the same country of this study, Korea, 1244 elementary school children were analyzed about the prevalence of bronchial hyper-responsiveness and its associated factors[35]. Contributing factors that increase the risk of bronchial hyper-responsiveness were younger age, higher eosinophil percent, lower FEV1, a fraction of exhaled nitric oxide (FeNO) ≥ 25ppb, and sensitization to HDM. It seems relatively clear that age and number of sensitized allergens will affect reducing bronchial hyper-responsiveness, however, the effect by SLIT is still unknown[20, 36].

## Conclusion

Three-year treatment of SLIT in Korean children with allergic rhinitis sensitized to HDM showed a decreased in eosinophil percent, which may suggest potential suppression effect on HDM sensitization. However, there was no effect of SLIT in terms of preventing new sensitization development and the increment of poly-sensitization rate, and improving bronchial hyper-responsiveness. In order to validate these results and to confirm the association with symptom change, further investigations with a larger cohort in multi-countries in Asia and more long-term follow-up will be required.
